# ROLE OF MACHINE LEARNING (ML) IN AGING IN PLACE RESEARCH: A SCOPING REVIEW

**DOI:** 10.1093/geroni/igae098.3890

**Published:** 2024-12-31

**Authors:** Sojung Park, Eunhye Ahn, Tae-Hyuk Ahn, SangNam Ahn, Soobin Park, Eunsun Kwon, Seoyeon Ahn, Yuanyuan Yang

**Affiliations:** Washington University in St. Louis, St. Louis, Missouri, United States; Washington University in St. Louis, St. Louis, Missouri, United States; Washington University in St. Louis, St. Louis, Missouri, United States; Saint Louis University, St. Louis, Missouri, United States; Washington University in St. Louis, St. Louis, Missouri, United States; Fairleigh Dickinson University, Hackensack, New Jersey, United States; Duke-NUS Medical School, Singapore, Singapore; Washington University in St. Louis, St. Louis, Missouri, United States

## Abstract

As global aging accelerates, Aging in Place (AIP) is increasingly central to improving older adults’ quality of life. Machine Learning (ML) is widely used in aging research, particularly in health monitoring and personalized care. However, most studies focus on clinical settings, leaving a gap in understanding ML’s application in non-clinical AIP contexts. This review addresses this gap by exploring the themes, policy implications, and ethical concerns of AIP-ML studies, including AI bias and fairness. The review examined 32 peer-reviewed studies sourced from databases like PsycINFO, MEDLINE, and PubMed. Through thematic analysis, three main themes emerged: successful aging, managing depressive symptoms, and fostering social connectedness. The studies employed various ML techniques, including classification algorithms, random forest, SVM, and LASSO, to predict health outcomes such as depression, cognitive decline, and functional disabilities in older adults. They also identified key risk factors and examined disparities in healthcare access and digital inclusion.However, the studies often lacked validation across diverse populations, which limits their policy impact. Most research focused on healthy, community-dwelling older adults, excluding those with dementia or disabilities, thereby introducing biases that could disproportionately affect marginalized groups The review highlights that while ML models are useful for prediction, they may overlook the needs of less healthy older adults, reducing their accuracy and fairness. These findings emphasize the need for transparency and participant involvement in data usage, suggesting that despite its limitations, the current literature offers valuable insights for advancing AIP research and guiding the development of more inclusive AIP policies.

